# *Streptomyces* sp. BI87 from human gut: potent anticancer activities and divergence from known *Streptomyces* lineages

**DOI:** 10.1128/spectrum.00858-25

**Published:** 2025-08-13

**Authors:** Yu-Hui Wang, Hong-Tao Xu, Miao-Wei Liu, Bao-Juan Yuan, Xin-Yu Gao, Xing-Hua Zhang, Hong-Da Tian, Hao Yu, Jin-Ru Lai, Liang Liu, Randal N. Johnston, Gui-Rong Liu, Shu-Lin Liu

**Affiliations:** 1 Genomics Research Center (Key Laboratory of Gut Microbiota and Pharmacogenomics of Heilongjiang Province, State-Province Key Laboratory of Biomedicine-Pharmaceutics of China), College of Pharmacy, Harbin Medical University 34707, Harbin, China; 2 Harbin Medical University-University of Calgary Cumming School of Medicine Center for Infection and Genomics, Harbin Medical University 34707, Harbin, China; 3 State Key Laboratory of Frigid Zone Cardiovascular Diseases, Harbin Medical University 34707, Harbin, China; 4 Department of Biochemistry and Molecular Biology, University of Calgary 2129, Calgary, Canada; 5 Department of Microbiology, Immunology and Infectious Diseases, University of Calgary 2129, Calgary, Canada; Ruhr-Universitat Bochum, Bochum, Germany

**Keywords:** natural species, anticancer, gut microbiota, polyphasic bacterial taxonomy

## Abstract

Cancer is among the deadliest diseases with few truly curative therapies. Recent studies have shown that the human intestinal microbiota contains bacteria that exhibit potent suppressive effects on cancer or coordinate anticancer functions with the immune system. However, whether the peculiar anticancer trait was acquired stochastically by a strain of a non-anticancer bacterial species or an intrinsic property of a phylogenetic lineage of bacteria remained unclear. In this study, we compared a human gut isolate *Streptomyces* sp. BI87, which had highly effective anticancer activities, with the type strain of *Streptomyces albidoflavus*, DSM 40455^T^, which was most closely related to BI87 among 30 type strains of the genus *Streptomyces* but did not show any detectable anticancer activities. We found that, although BI87 and DSM 40455^T^ had >99% 16S rDNA sequence similarity, they are phylogenetically separated by a genetic boundary into distinct natural species as evaluated by the 3Cs definition of bacterial species based on the ratio of common genes with zero sequence degeneracy. In addition to the anticancer activities, BI87 encodes several biological characteristics different from DSM 40455^T^, including those of antibacterial and antiviral spectra, secondary metabolites, structural composition in the cell wall, and major cellular fatty acids. Based on the genomic and phenotypic distinctions of BI87 from closely related *Streptomyces* strains, we propose that the anticancer bacterial strain BI87 represents a new lineage at the natural species level within the genus *Streptomyces*.

**IMPORTANCE:**

The characterization of the anticancer bacterial strain, *Streptomyces sp*. BI87, isolated from a healthy human, suggests the prevalent existence of anticancer microbes in the gut microbiome of humans, which may be nurtured and harnessed for cancer prevention or treatment without the need for the introduction of engineered and non-indigenous microbes to a person or the use of radio- or chemo-therapies. Also important is the finding that a close relative of BI87, *Streptomyces albidoflavus* DSM40455^T^, does not express appreciable anticancer properties in the same *in vitro* and *in vivo* experiments, demonstrating that *Streptomyces *sp. BI87 represents a novel bacterial lineage with selective suppressive activities on cancer, at the phylogenetic level of natural species. Genomic comparisons between BI87 and DSM40455^T^ demonstrate that phylogenetic delineation of closely related bacteria needs to be conducted at the level of natural species rather than OTUs, as an OTU may contain phenotypically or even phylogenetically radically distinct bacteria.

## INTRODUCTION

Cancer is among the deadliest diseases with few truly curative therapies. Recent studies have shown that the human intestinal microbiota contains bacteria that exhibit potent suppressive effects on cancer or coordinate anticancer functions with the immune system, such as those of the genus *Streptomyces*, demonstrating a potentially novel way of cancer control that is curative without causing adverse reactions (1–8). The bacterial genus *Streptomyces*, first described in 1943, belongs to the phylum Actinomycetota (previously called Actinobacteria) and encompasses an enormously diverse group of gram-positive bacteria with high G + C contents (69%–73%; mol%) (9–12). Many *Streptomyces* species are known to produce various compounds of significant health value, and to date, over 60% of the microbial substances in medical application are derived from *Streptomyces*, including anticancer agents (13), antibiotics (14), immunosuppressants (15), herbicides (16), antioxidants (17), and new siderophores (18). More than 500 antibiotics are secondary metabolites or derivatives of *Streptomyces* (19).

Closely related bacteria may have radically different biological properties (20). For example, *Streptomyces* strains isolated from fecal samples may or may not have anticancer effects, even though they could be assigned to the same groupings based on genomic relatedness (6). This would inevitably pose a critical question: Are anticancer properties strain-unique or species-specific? If the former, the isolation of an anticancer strain will have extremely scarce chances, and the traits may be easily lost; if the latter, the anticancer traits may be more stable, and the evolution of such anticancer traits may be investigated systematically according to phylogeny based on the assumption that strains of the same natural species may share most of the biological characteristics, including, for instance, anticancer activities. However, the “natural species” concept will have to be unambiguously defined in the first place based on truly natural rather than arbitrary parameters (21–23).

The current bacterial taxonomy defines species based on two requirements: ≥97% 16S rRNA gene sequence identity and ≥70% DDH (DNA-DNA hybridization) values between bacterial strains in comparison (24–27). However, both parameters are continuous without clear-cut boundaries to separate closely related bacteria into well-isolated and non-overlapping clusters at the level equivalent to natural species. As a result, species so defined may be a mixture of phenotypically and, especially, biologically different bacteria (28, 29). In previous studies, we employed *Salmonella* as primary models to delineate bacteria into discrete phylogenetic units by three criteria and proposed to use the three criteria in defining a natural species as follows: members of a natural species should have exclusively close relatedness, keep clear-cut genetic boundaries with bacteria of other natural species, and share common biological features; we call the three criteria the 3Cs concept of natural bacterial species (22). Based on the 3Cs concept and the parameters, the 2,600-plus *Salmonella* lineages could be classified into individual natural bacterial species (20, 21, 23). Our research has also demonstrated that the bacterial speciation process involves a series of molecular events, including genetic boundary formation coordinated by the genetic switch mechanism, to isolate nascent natural bacterial species into distinct micro-niches or gene pools (30–33).

Previously, we reported a gut bacterial strain, *Streptomyces* sp. BI87, which had a potent anticancer activities (6), but its phylogenetic position has remained undetermined. In this study, we made genomic comparisons between strain BI87 and the type strains of known *Streptomyces* species. We found that strain BI87 was most closely related to the type strain of *Streptomyces albidoflavus*, DSM 40455^T^, but there was a clear-cut genetic boundary between them, suggesting BI87 to be a representative of a new *Streptomyces* lineage (21, 23). Importantly, BI87 and DSM 40455^T^ exhibited significant distinction in biological properties. Although strain BI87 had a potent anticancer effect, *S. albidoflavus* did not, consistent with the 3Cs concept to circumscribe closely related bacteria into discrete natural bacterial species (22). We also conducted polyphasic classification processes, which were also supportive of the phylogenetic as well as phenotypic distinction of BI87 from other *Streptomyces* lineages. In establishing *Streptomyces* sp. BI87 as a novel species, we have focused our comparative analyses primarily on its closest phylogenetic relatives. This approach is standard practice in bacterial taxonomy because demonstrating clear and consistent differentiation from the most similar existing species provides the strongest evidence for novelty. While anticancer activity may exist in other, more distantly related bacteria, the key to defining a new species lies in establishing a distinct evolutionary lineage with its own unique set of characteristics, particularly when compared to its closest neighbors in the phylogenetic tree. This study provides novel evidence showing that commensals in the gut microbiome may have potent anticancer activities independent of coordination with the immune system, although their synergy may play even more radical roles in the body against malignancy. Also important is BI87, as a precious example to support the 3Cs criteria in delineating closely related bacteria into discrete phylogenetic lineages equivalent to natural species (21–23), by highlighting phenotypic distinction, specifically anticancer activity.

## RESULTS

### Strain BI87 is most closely related with *S. albidoflavus* DSM 40455^T^

**Fig 1:**
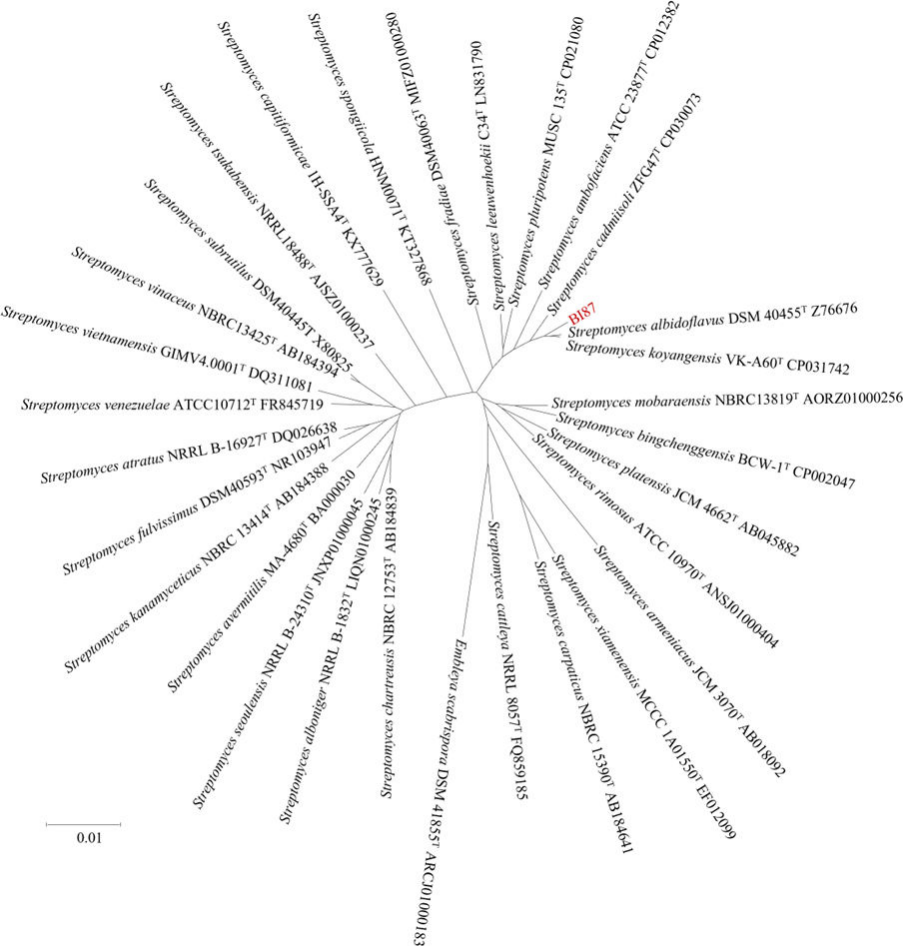
Neighbor-joining tree based on 16 rRNA gene sequences showing the position of strain BI87 among its phylogenetic neighbors. *Embleya scabrispora* DSM 41855^T^ was used as an outgroup. GenBank accession numbers are given following the strain numbers. Bar, 0.01 substitutions per site.

To determine the phylogenetic position of strain BI87 and estimate its evolutionary relationships with known *Streptomyces* species, we constructed a 16S rDNA sequence tree (Fig. 1). We found that strain BI87 exhibited closest relatedness to *S. albidoflavus* DSM 40455^T^ with 99.44% 16S rDNA sequence similarity, followed by *Streptomyces koyangensis* VK-A60^T^ (99.10%). According to the current bacterial species definition (34), if 16S rRNA gene sequence similarity between two strains is ≥97%, DDH (≥70% for bacteria of the same species) or average nucleotide identity (ANI; ca. 95%–96% being considered equivalent to a DDH value of 70%) values will be needed for further evaluating the phylogenetic relationships of the bacteria compared (35–39). Genomic comparisons of strain BI87 with *S. albidoflavus* DSM 40455^T^ showed a DDH value of 81.80% and an ANI value of 95.93%, which are both consistent with the 16S rDNA result between them. These values would allow the assignment of strain BI87 to the *S. albidoflavus* species according to the current taxonomic criteria for species definition. However, as the anticancer property is a key biological trait of bacteria as seen in BI87, we cannot ignore it while evaluating the phylogenetic relationships between BI87 and *S. albidoflavus* DSM 40455^T^, especially while determining whether they might belong to the same natural species according to the 3Cs definition (21–23).

### Potent anticancer activities in strain BI87 but not in *S. albidoflavus* DSM 40455^T^

**Fig 2:**
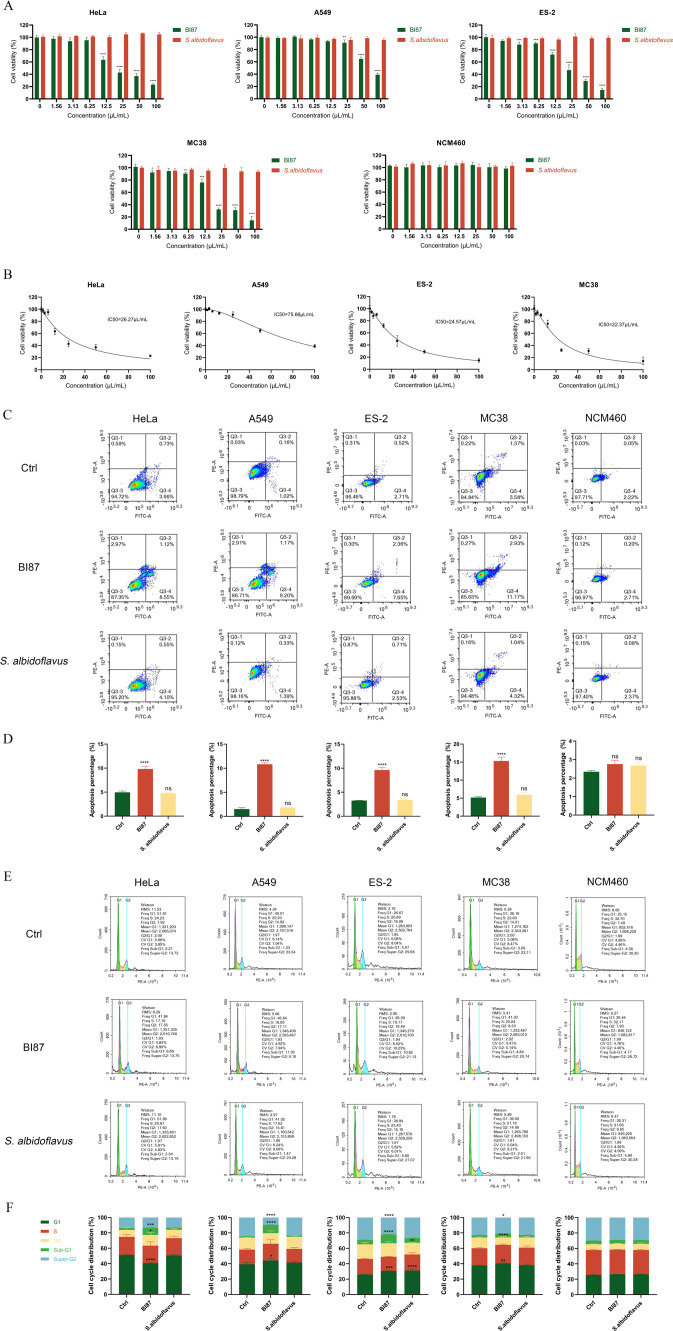
Suppressive effects of BI87 on cancer cell lines in comparison with *S. albidoflavus* DSM 40455^T^. (**A**) Dose dependence of T effects of BI87 and *S. albidoflavus* DSM 40455^T^ culture on HeLa, A549, ES-2, MC38, and NCM460 by CCK8 assay. (**B**) IC50 determination of BI87 fermentation supernatant on HeLa, A549, ES-2, and MC38 cell viability. (**C**) The apoptosis induced by BI87 and *S. albidoflavus* DSM 40455^T^ culture detected by flow cytometry. (**D**) Quantitative analysis of apoptotic cells. (**E**) Flow cytometry was used to analyze the cell cycle distribution of cells treated with BI87 and *S. albidoflavus* DSM 40455^T^ culture. (**F**) The results of the cell cycle were evaluated statistically. Data were presented as mean ± SD of at least three independent experiments. ns *P* > 0.05, *P < 0.05, **P ≤ 0.01, ***P ≤ 0.001, and ****P < 0.0001 vs control.

We had previously validated the potent anticancer activities of BI87 by both *in vitro* and *in vivo* experiments (6). Here, we compared BI87 and *S. albidoflavus* DSM 40455^T^ for their effects on cancer cell lines. Consistent with the previous report (6), strain BI87 showed potent suppressive effects on cervical cancer HeLa, non-small cell lung cancer A549, ovarian clear cell carcinoma ES-2, and colorectal cancer MC38 cell lines, whereas *S. albidoflavus* DSM 40455^T^ did not exhibit suppression on any of these cancer cell lines (Fig. 2A), demonstrating their radical differences in biological behaviors. To determine whether BI87 supernatant was selectively toxic to cancer cells, we included the non-cancerous cell line NCM460 and did not find appreciable cytotoxic effects, demonstrating that BI87 selectively suppresses cancer cells but not non-cancerous cells (Fig. 2A). The IC50 values of BI87 supernatant in HeLa, A549, ES-2, and MC38 cells were 26.27, 75.66, 24.57, and 22.37 µL/mL, respectively (Fig. 2B). When the concentration of BI87 reached or exceeded 50 µL/mL, it exhibited the most potent cytotoxic effects against these cancer cell lines. Therefore, 50 µL/mL was used as the optimal concentration for subsequent experiments.

To elucidate the possible mechanism underlying the inhibitory effects of BI87 on cancer cell proliferation, we detected the level of apoptosis after treatment with the bacterial culture supernatant. The cells were double stained with Annexin V and PI to quantitatively analyze the apoptotic effects by flow cytometry. As shown in Fig. 2C and D, after the addition of BI87 supernatant, the percentages of early and late apoptotic HeLa, A549, ES-2, and MC38 cells increased, indicating that BI87 killing involves apoptosis. Analysis of the cell cycle distribution via flow cytometry revealed that BI87 treatment increased the proportion of cells in the G2 phase and induced apoptosis (Sub-G1 population; Fig. 2E and F), suggesting DNA damage or mitotic disruption. In contrast, *S. albidoflavus* DSM 40455^T^, which lacks cytotoxic activity, caused reversible G2 arrest in MC38 cells without apoptosis, likely due to transient cell cycle checkpoint activation without lethal damage.

### Genomic sequence of strain BI87 and its comparisons with closely related *Streptomyces* strains

**Fig 3:**
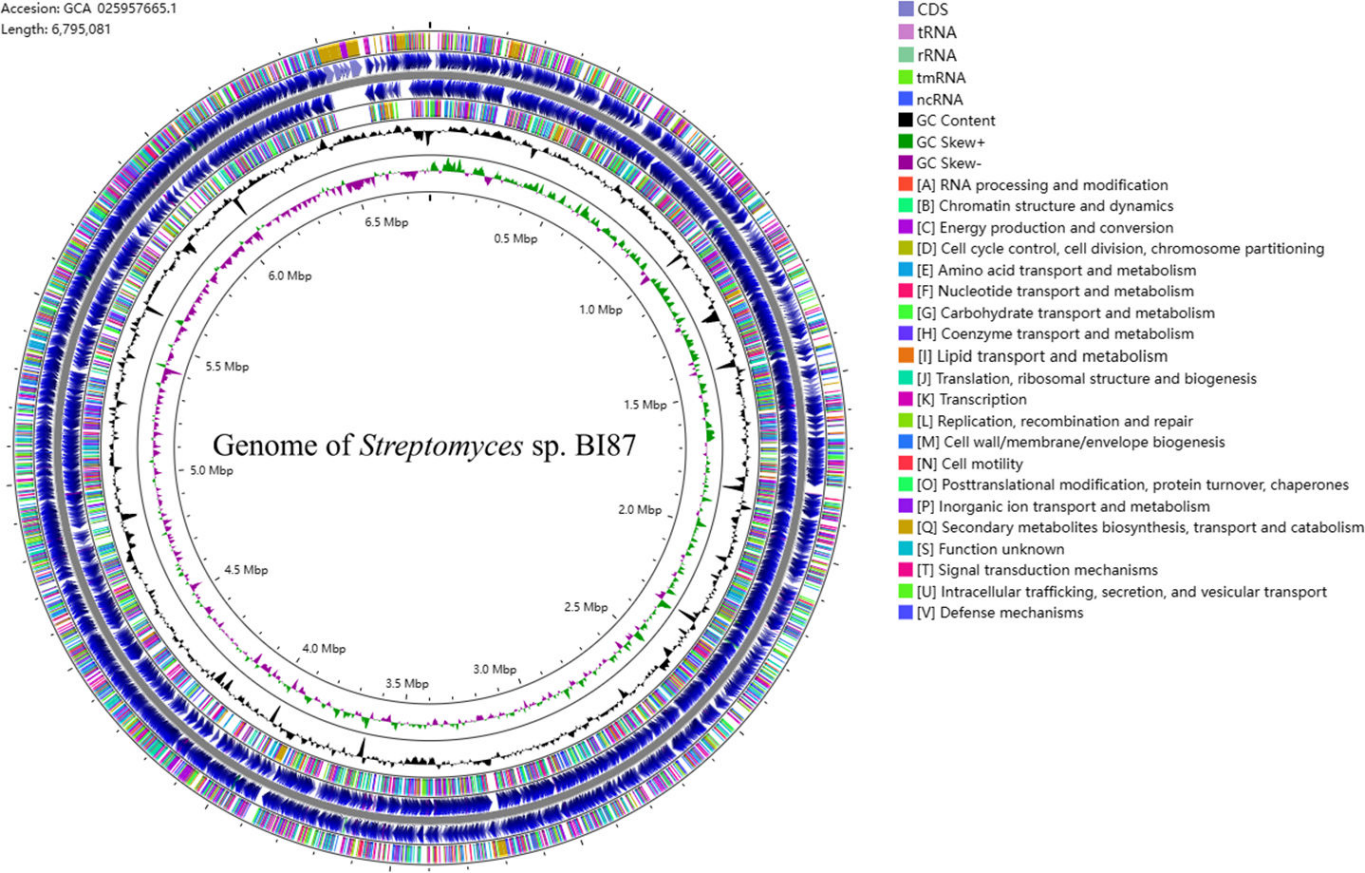
Genome map of strain BI87. From outer to inner rings: GC skew± (strand bias), GC content, functional COG categories (highlighting [Q] secondary metabolites and [J] translation), and genomic features (CDS/tRNA/rRNA).

The full or null anticancer activities between BI87 and *S. albidoflavus* DSM 40455^T^ made it necessary to reveal the genomic distinctions of BI87 from *S. albidoflavus* DSM 40455^T^ that have made the closely related bacteria so different in biological properties. We therefore conducted systematic genomic comparisons between strain BI87 and closely related *Streptomyces* strains, including *S. albidoflavus* DSM 40455^T^ and five additional *S. albidoflavus* strains (CP064783, CP040466, CP059254, NC020990, and CP014485), as well as *S. koyangensis* VK-A60^T^ and an additional *S. koyangensis* strain (CP049945). The BI87 genome is a single closed circle, 6,795,081 bp in size, with a GC content of 73.50%, 6,306 coding sequences, 64 tRNA genes, and 21 rRNA genes (Fig. 3). We extracted genes common to BI87 and the previously sequenced *Streptomyces* strains and profiled sequence variations among them. As shown in Table S1, genes common to BI87 and the type strains of *S. albidoflavus* (DSM 40455^T^) and *S. koyangensis* (VK-A60^T^) were 83% or lower. Within *S. albidoflavus* or *S. koyangensis*, the situations were different. *S. albidoflavus* DSM 40455^T^ had 89%–94% common genes with three of the five additional *S. albidoflavus* strains in the comparison, CP059254, NC020990, and CP014485, but only 83%–86% with the other two *S*. *albidoflavus* strains, CP064783 and CP040466; the two *S*. *koyangensis* strains, VK-A60^T^ and CP049945, shared up to 92% of their genes. Interestingly, the two *S*. *albidoflavus* strains with lower percentages of common genes with *S. albidoflavus* DSM 40455^T^, i.e., CP064783 and CP040466, shared much higher percentages of common genes with BI87 (up to 94%) than with the type strain DSM 40455^T^ (up to 86%).

### Detection of the genetic boundary between strain BI87 and strains of *S. albidoflavus* and *S. koyangensis*

**Fig 4:**
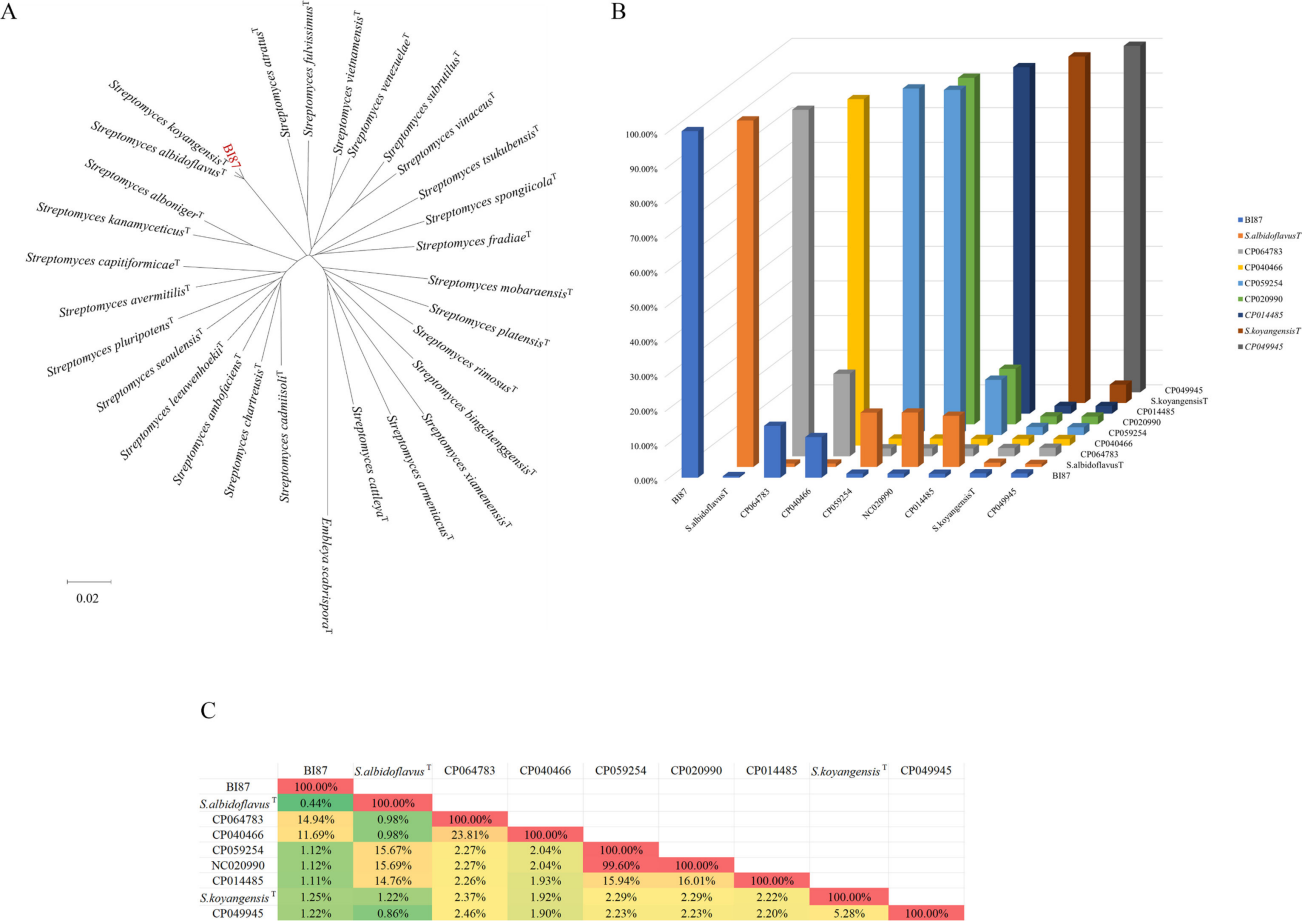
Genomic comparison among the *Streptomyces* strains. (**A**) Neighbor-joining tree for 30 sequenced *Streptomyces* strains, based on whole-genome sequences (all conserved regions among the compared genomes are concatenated and aligned for tree construction). *Embleya scabrispora* DSM 41855^T^ was used as an outgroup. Bar, 0.02 substitutions per site. (B) Sequences common to all nine strains were concatenated and pair-wise aligned for the number of genes that have identical sequences (zero nucleotide degeneracy). (C) Ratios of common genes with zero nucleotide degeneracy between pairs of strains compared.

To determine whether BI87 may have diverged enough from closely related *Streptomyces* lineages to represent a nascent species, we detected genetic boundaries between BI87 and closely related *Streptomyces* strains. For this, we concatenated genes common to BI87 and the type strains of 30 taxonomic species of *Streptomyces* and built a core genome tree (Fig. 4A). The phylogenetic relationships between BI87 and the *Streptomyces* type strains reflected by the core genome tree were consistent with those of the 16S rDNA tree (see Fig. 1), with BI87 most closely related to *S. albidoflavus* and *S. koyangensis*.

We then focused on the comparisons of BI87 with strains of *S. albidoflavus* and *S. koyangensis* to determine the ratios of genes with zero sequence degeneracy (genes with identical sequences without any nucleotide variation) between pairs of strains in comparison (20, 21, 23). Of great significance, the ratios were lower than 2% both between BI87 and *S. albidoflavus* DSM 40455^T^ and between BI87 and *S. koyangensis* VK-A60^T^ (Fig. 4B and C). Such ratios are similar to the 6% ratio between *Salmonella typhi* and *Salmonella typhimurium* (23). As the 6% ratio of zero degeneracy genes divided *S. typhi* and *S. typhimurium* into separate natural species, the <2% ratio of zero degeneracy genes would demonstrate the existence of genetic boundaries to separate BI87 from *S. albidoflavus* DSM 40455^T^ and from *S. koyangensis* VK-A60T at the level of natural species.

### Genomic divergence of BI87 from *S. albidoflavus* and *S. koyangensis*

**Fig 5:**
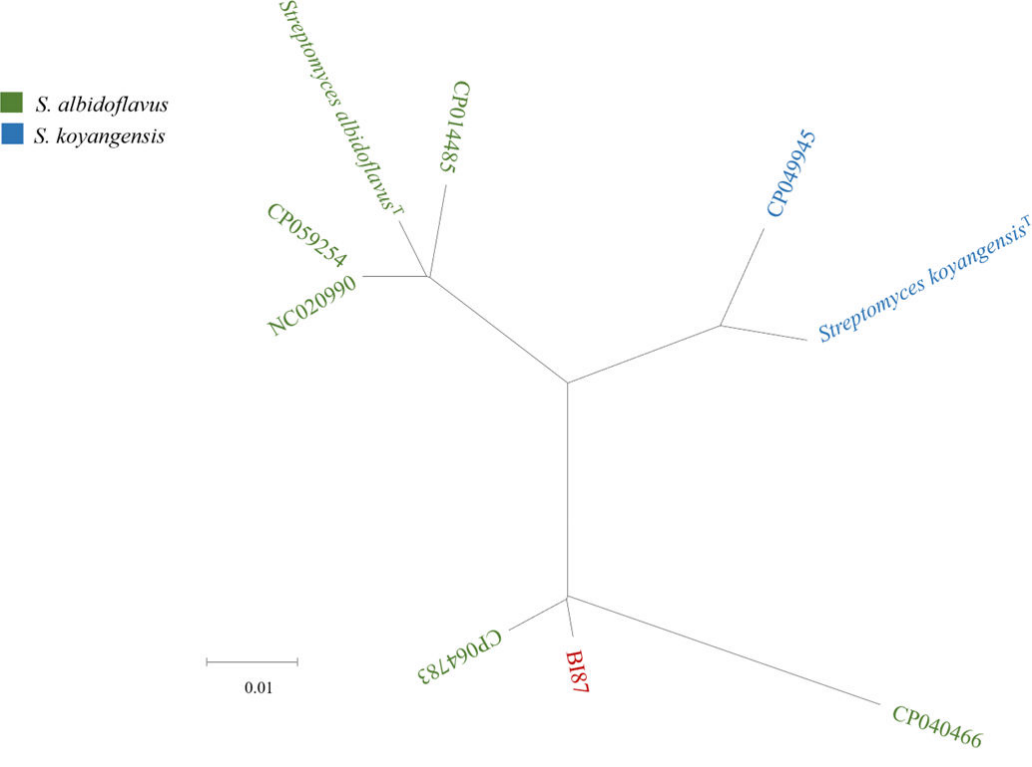
Phylogenetic relationships of BI87 with closely related strains of *S. albidoflavus* and *S. koyangensis* based on conserved sequences. Bar, 0.01 substitutions per site. GenBank accession numbers are given following the strain numbers. Note that *S. albidoflavus* CP064783 and CP040466 are more closely related to BI87 than to the type strain of *S. albidoflavus*, DSM 40455^T^, and the other *S. albidoflavus* strains compared.

To reveal the overall divergence of BI87 from *S. albidoflavus* and *S. koyangensis*, we constructed a core genome phylogenetic tree for the analysis (Fig. 5). The tree divided the nine strains into three well-separated clusters, including the *S. albidoflavus* cluster of four strains (DSM 40455^T^, CP059254, NC020990, and CP014485), the *S. koyangensis* cluster of two strains (VK-A60^T^ and CP049945), and the BI87 cluster of three strains (BI87 and two *S*. *albidoflavus* strains, CP064783 and CP040466), consistent with the percentages of common genes among them (see Table S1) and the ratios of genes with zero degeneracy between pairs of the strains (see Fig. 4). This result would allow *S. albidoflavus* CP064783 and CP040466 to be categorized with BI87, especially considering the higher ratios of zero degeneracy genes of CP064783 and CP040466 with BI87 (14.94% and 11.69%, respectively; see Fig. 4C) than with their taxonomic type strain DSM 40455^T^ (both being 0.98%).

### Functional annotation of the BI87 genome

**Fig 6:**
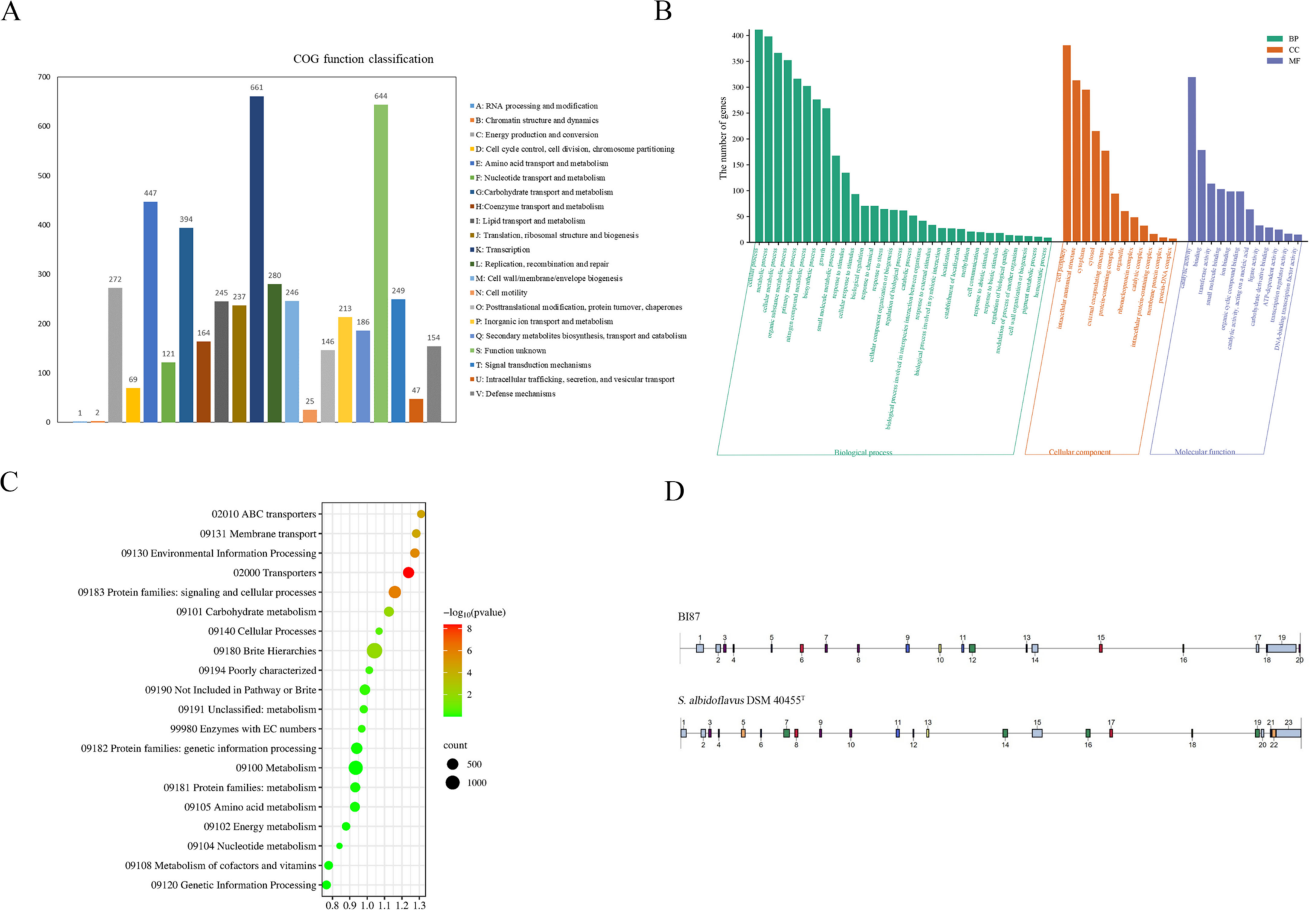
Functional annotation of the BI87 genome. (**A**) The predicted functions by the COG database search and analysis. (**B**) Functions predicted by the GO database search and analysis. (**C**) Predictions and analysis by KEGG database searches. (**D**) Comparison of genomic loci of the biosynthetic gene clusters between BI87 and *S. albidoflavus* DSM 40455^T^.

To predict functional properties of BI87, we deduced the proteins encoded by the genome using the eggNOG database and annotated a total of 5,675 coding sequences. According to the prediction results of the COG database, we categorized 5,646 genes into 21 functional groups, with genes in amino acid transport and metabolic pathways having the highest number of transcripts, followed by genes in unknown function, amino acid transport and metabolism, carbohydrate transport and metabolism, and others (Fig. 6A).

The results of GO annotation of the BI87 genome (Fig. 6B) showed the highest number of genes involved in biological processes (BP; 3,731 genes), followed by cellular components (CC; 1,635 genes) and molecular function (MF; 1,073 genes). The top three annotated BPs include cellular processes (411 genes), metabolic processes (398 genes), and cellular metabolic processes (366 genes); the top three CCs include the cell periphery (380 genes), cell membrane compositions (312 genes), and intracellular anatomical structure and cytoplasm (294 genes); and the top three MFs include catalytic activities (318 genes), binding (177 genes), and transferase activities (112 genes). A total of 1,474 genes were annotated to KEGG pathways in BI87, from which the top 20 pathways with the highest numbers were displayed (Fig. 6C). Among them, the highest numbers of genes were enriched to brite hierarchies (1,394 genes), metabolism (1,052 genes), signaling and cellular processes (669 genes), and genetic information processing (511 genes).

In terms of resistance prediction, BI87 had all resistance genes that *S. albidoflavus* DSM 40455^T^ had except fusidic acid and nucleoside antibiotics (Table 1). The antiSMASH analysis showed that the genome of BI87 contains various secondary metabolite biosynthetic gene clusters (BGCs), including non-ribosomal peptide synthetases (NRPs), polyketide synthases, ribosomally synthesized and post-translationally modified peptides (RiPPs), and other antimicrobial synthases (Fig. 6D; Table S2). BI87 contains the lassopeptide gene cluster, encoding a unique family of natural products that have been reported to have antibacterial, anticancer, and anti-HIV activities (40–42). The discovery of these compounds (Table 2) also provides an important basis for suggesting that the BI87 diverged from the *S. albidoflavus* DSM 40455^T^ clade and evolved independently into a new lineage with new traits.

**TABLE 1:** The numbers of drug resistance genes of strain BI87

Drug class	BI87	*S. albidoflavus*
Acridine dye	9	18
Aminocoumarin antibiotic	26	41
Aminoglycoside antibiotic	19	59
Bicyclomycin	1	1
Carbapenem	8	62
Cephalosporin	26	93
Cephamycin	15	60
Diaminopyrimidine antibiotic	2	44
Elfamycin antibiotic	3	3
Fluoroquinolone antibiotic	37	133
Fosfomycin	7	12
Glycopeptide antibiotic	19	21
Glycylcycline	6	20
Isoniazid	2	12
Lincosamide antibiotic	23	21
Macrolide antibiotic	103	140
Monobactam	8	41
Mupirocin	2	6
Nitroimidazole antibiotic	11	6
Oxazolidinone antibiotic	19	11
Penam	45	131
Penem	4	62
Peptide antibiotic	19	73
Phenicol antibiotic	34	106
Pleuromutilin antibiotic	21	17
Rifamycin antibiotic	16	39
Streptogramin antibiotic	25	20
Sulfonamide antibiotic	3	18
Tetracycline antibiotic	87	186
Fusidic acid	0	2
Nucleoside antibiotic	0	4
Triclosan	9	29

**TABLE 2:** Comparative secondary metabolite biosynthesis gene of BI87 with its closely related species^*a*^

Region	Type	Location	Most similar known cluster	Similarity
Region 9	Lanthipeptide-class-iii	2,838,061–2,860,640	AmfS	RiPP:lanthipeptide (100%)
Region 10	Lassopeptide	3,087,848–3,110,135		
Region 11	NRPS	3,173,470–3,233,316	Dechlorocuracomycin	NRP (16%)
Region 12	RiPP-like	3,794,238–3,803,789	Herbimycin A	Polyketide (6%)

^*a*^ The empty cells indicate that no known biosynthetic gene cluster (BGC) with significant similarity was identified in the reference databases, highlighting a potentially unique biosynthetic pathway in this strain.

### Polyphasic characterization of strain BI87

**Fig 7:**
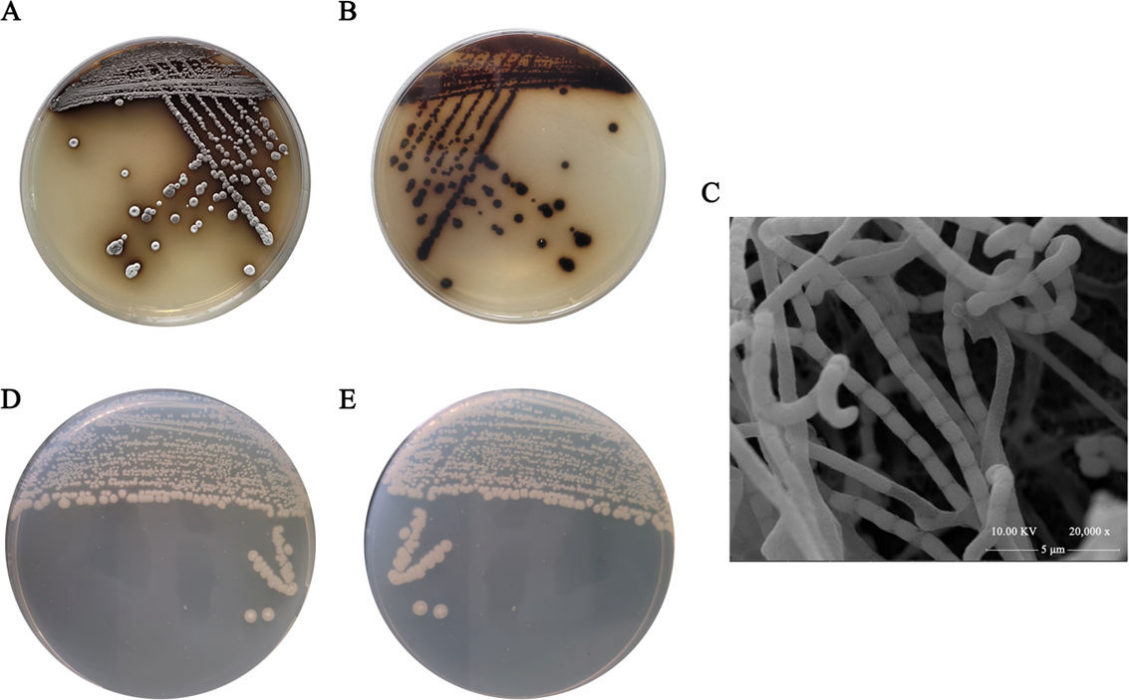
Comparisons of colony morphology on Gause’s Synthetic agar media plate between BI87 and *S. albidoflavus* DSM 40455^T^. (**A**) Morphological photograph of strain BI87 on the front of the plate. (**B**) Morphological photograph of strain BI87 on the back of the plate. (**C**) Scanning electron micrograph of BI87. (**D**) Morphological photograph of strain *S. albidoflavus* DSM 40455^T^ on the front of the plate. (**E**) Morphological photograph of strain *S. albidoflavus* DSM 40455^T^ on the back of the plate.

To further demonstrate the existence of the hypothesized genetic boundary between BI87 and *S. albidoflavus* DSM 40455^T^, we carried out polyphasic taxonomical studies for their comparison. Strain BI87 grew well on NA, R_2_A, Gause’s synthetic No. 1 medium, and *Streptomyces* Project (ISP) 1–5 agar media. On Gause’s synthetic No. 1 agar plates, the colonies formed brown substrate hyphae and abundant white to gray aerial hyphae and produced brown-green pigment (Fig. 7A and B). The aerial mycelium of BI87 was divided into straight or curved chains of spores (Fig. 7C). Each spore was ovoid to cylindrical with a smooth and inactive surface. In contrast, the aerial mycelia of *S. albidoflavus* DSM 40455^T^ proliferated well on most of the ISP1-5 culture media, with the substrate mycelia and aerial mycelia being in white color without soluble pigments (Fig. 7D and E).

For BI87, the growth temperature range was 25°C–40°C (optimum, 37°C), and the pH range was pH 5–12 (optimum, pH 7). NaCl tolerance of BI87 was up to 4% (wt/vol). Strains BI87 and *S. albidoflavus* DSM 40455^T^ differed in their utilization of carbon sources (Table S3), such as D-turanose, α-D-lactose, D-melibiose, D-salicin, and N-acetyl-D-glucosamine. In addition, differences in the enzymatic activities of the two strains, including α-galactosidase, also helped to distinguish the isolates from their related species.

### Chemotaxonomic characterization

**Fig 8:**
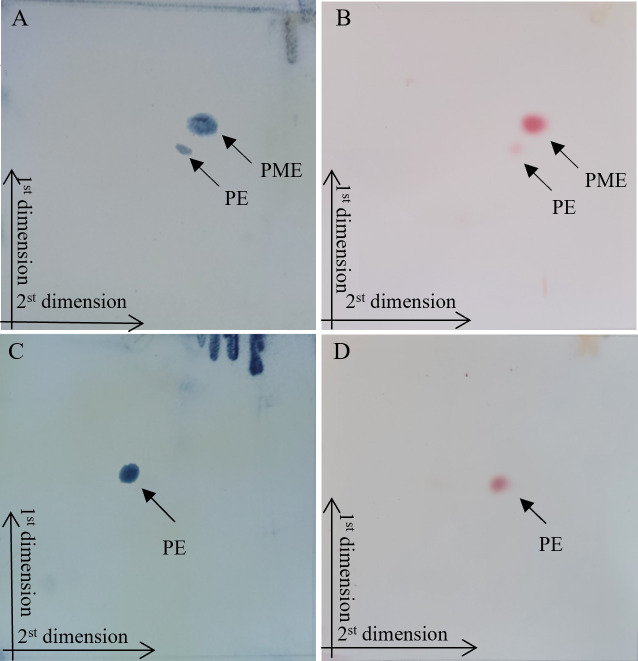
Two-dimensional TLC of the total polar lipids of strain BI87 and *S. albidoflavus* DSM 40455^T^ stained with ninhydrin chromogenic agent (**A and C**) and Dittmer and Lester agent (**B and D**).

The chemotaxonomic studies confirmed that strain BI87 exhibited characteristics typical of members of the genus *Streptomyces*. The cell wall of BI87 contained LL-diaminopimelic acid. Whole-cell hydrolysates consisted of ribose and xylose for strain BI87, while *S. albidoflavus* DSM 40455^T^ contained ribose but not xylose. The polar lipid profile of strain BI87 consisted of phosphatidylethanolamine (PE) and phosphatidylmonomethylethanolamine (PME), whereas *S. albidoflavus* DSM 40455^T^ contained PE but not PME (Fig. 8). The major cellular fatty acids (>10%) of strain BI87 were anteiso-C15:0, C16:0, iso-C16:0, and anteiso-C17:0 (Table S4), whereas those of *S. albidoflavus* DSM 40455^T^ were iso-C15:0, anteiso-C15:0, and C16:0. The major menaquinones of strain BI87 consisted of MK-9 (H6) and MK-9 (H8), which are also typical of members of the genus *Streptomyces*. These results demonstrated considerable biological divergence of BI87 from *S. albidoflavus* DSM 40455^T^.

## DISCUSSION

The prevalent existence of anticancer bacteria in the human gut opens great opportunities for making use of such bacteria in the treatment of malignant diseases as curative, rather than merely alleviative, therapies without causing adverse side reactions often seen in chemotherapies. Two strategies for using such bacteria can be explored. One is to use the bacterial anticancer products in the culture supernatant or the anticancer substance purified from the bacterial culture. The second strategy is to nourish the natural anticancer bacteria in the gut by ameliorating the disordered microenvironment of the gut. In either strategy, it is necessary to know who is producing the anticancer substances and whom we should nourish to have the anticancer substances produced.

First of all, there is evidence showing that anticancer bacteria are normal residents in the human gut (6). Therefore, it is reasonable to postulate that cancer can hardly be formed in a person unless the individual has a genetic predisposition (BRCA1/2 or other gene defects), exposes environmental risks such as cigarette smoke, or suffers from gut dysbiosis due to unhealthy lifestyles or antimicrobial abuse. Indeed, cancers are closely associated with gut dysbiosis (43), and amelioration of the disordered gut microbiome by administering certain natural compounds could receive curative effects (7). How can the gut microbiome be ameliorated? Currently, there is no definite answer to such a question, as the current bacterial taxonomy does not classify the bacteria down to the level of natural species yet. The current taxonomic species of bacteria, usually taken as equivalent to operational taxonomy units (OTUs), in fact, often categorizes phylogenetically much more diverse bacteria than natural species into an OTU, as demonstrated by distinct lineages of *Escherichia coli*, which could be delineated into distinct phylogenetic groupings equivalent to natural species (5). Previously, we proposed the 3Cs criteria to delineate bacteria into natural species (21–23) and used the parameters to define the natural bacterial species (20). In this study, we compared the gut bacterial strain *Streptomyces* sp. BI87 that has potent anticancer activities with very closely related *Streptomyces* strains and found that *Streptomyces* sp. BI87 had clear-cut genetic boundaries with the type strains of 30 presently known *Streptomyces* species. Therefore, we conclude that *Streptomyces* sp. BI87 represents a novel *Streptomyces* species, and its exceptional biological property is the anticancer activities.

Additional properties of *Streptomyces* sp. BI87 are also supportive to our conclusion and indicate that the novel branch of *Streptomyces* represented by BI87 is a product of complex evolutionary processes, as it would be highly unlikely for so many peculiar traits to be acquired by a *Streptomyces* strain via a single stochastic genomic event at once, especially considering that the formation of genetic boundaries would take long periods of time. To gain a close-up view of genomic differences of BI87 from other *Streptomyces* lineages, we focused on the comparisons between strain BI87 and DSM 40455^T^, which is most closely related to BI87 among all *Streptomyces* strains compared and is the type strain of *S. albidoflavus*. In addition to the full or null anticancer property in BI87 or DSM 40455^T^, these two strains also differ in antibacterial and antiviral spectra, secondary metabolites, whole cell sugars, and major cellular fatty acids. Since antimicrobial resistance genes are important traits for adaptation in evolution (44–46), such differences also reflect divergence of the bacteria into distinct lineages.

*Streptomyces* have extremely strong ecological adaptabilities due to their enormous secondary metabolic capacities, acquired through vertical inheritance or horizontal gene transfer. The well-developed, diverse biosynthetic pathways make *Streptomyces* a great treasure trove of natural products. Selective pressure from the ecosystem promotes the migration of biosynthetic gene clusters and consolidates their presence in *Streptomyces* as core genetic traits to be stably maintained by vertical inheritance and highly conserved in the *Streptomyces* species (47–49). Interestingly, we predicted a special class of products, lasso peptides, encoded by a genomic island in the secondary metabolites of BI87, which are involved in host immunity enhancement and antimicrobial activities (50). How the lasso peptides have facilitated the evolution and speciation of a novel *Streptomyces* lineage represented by BI87, and especially how they made BI87 an excellent anticancer bacterial strain without adverse side effects, opens an invaluable area of research for curative therapies to control cancer.

It is possible that other *Streptomyces* species, both closely and distantly related to *Streptomyces* sp. BI87, may also possess anticancer activity. Future research should focus on systematically screening a wider range of bacterial species to identify novel anticancer agents and to understand the evolutionary distribution of this trait. Further studies are also needed to assess how these factors interact to promote or inhibit the observed anticancer effects.

### Conclusion

Strain BI87 has potent suppressive effects on a broad range of cancers and represents a novel *Streptomyces* lineage genetically separated by clear-cut boundaries from the type strains of currently known *Streptomyces* species. In addition, phenotypic, whole genome-based analyses and chemotaxonomic characterization were all supportive to indicate that strain BI87 represents a novel species.

## MATERIALS AND METHODS

### Bacterial strain isolation and collection

Strain BI87 was isolated from a human fecal specimen in Harbin city, China. The isolate was maintained on Gause’s agar media at 4°C and in 50% (vol/vol) glycerol at −80°C. *S. albidoflavus* DSM 40455^T^ was obtained from Shanghai Bioresource Collection Center. The bacterial strains were grown under the same conditions for comparative testing.

### Whole-genome sequencing

Strain BI87 was inoculated into Gause’s Synthetic liquid medium and cultured at 37°C for approximately 7 days at 150 rpm. The cell biomass was harvested after 2 min centrifugation at 12,000 rpm/min. Genomic DNA of strain BI87 was extracted using the Wizard Genomic DNA Purification Kit (Promega Corporation, USA). Purified genomic DNA was quantified by TBS-380 fluorometer (Turner BioSystems Inc., Sunnyvale, CA). Whole-genome sequencing was performed using a hybrid sequencing approach on the Illumina NovaSeq6000 and Nanopore Prometh ION platforms (Majorbio, Inc., China). DNA samples were sheared into 400–500 bp fragments using a Covaris M220 Focused Acoustic Shearer, purified, end-repaired, and ligated with sequencing adapters to construct a library. Sequencing reads were assembled by SOAP denovo v1.05 software (with *K* value set to 37) and SOAP aligner v2.21 software, and the splicing information of reads was counted, and single-base proofreading was performed on the assembly results. The reads matching information was used to calculate the assembly quality of Scaffold sequences and analyze the positional relationship between Scaffolds. Protein-coding sequences of each genome were predicted and annotated with Prokka v1.14.6 (51). The sequenced genome of BI87 was deposited in NCBI GenBank Database under BioProject PRJNA891902 with the accession number CP110079. The whole-genome sequence data reported in this paper have been deposited in the Genome Warehouse (52) in the National Genomics Data Center (53), Beijing Institute of Genomics, Chinese Academy of Sciences/China National Center for Bioinformation, under accession number GWHFHGG01000000 that is publicly accessible at https://ngdc.cncb.ac.cn/gwh.

### Phylogenetic analysis and molecular studies

16S rRNA gene sequences were obtained from the whole genome using barrnap v0.8 and annotated using EzBioCloud server (https://www.ezbiocloud.net/) (54). Digital DNA-DNA hybridizations were performed using the webserver Genome-to-Genome Distance Calculator version 2.1 (55), with Basic Local Alignment Search Tool (BLAST) as local alignment tool, as recommended. The ANI values were calculated using ChunLab’s online ANI calculator (56).

### Cell lines and cytotoxic activity assays

Single colonies of strain BI87 and *S. albidoflavus* DSM 40455^T^ were picked and inoculated in Erlenmeyer flasks (250 mL) containing 100 mL Gause’s Synthetic liquid medium and incubated at 37°C and 150 rpm in a rotary shaker incubator. After 9 days, the bacterial culture was centrifuged at 12,000 rpm for 2 min, and the supernatant was filtered through a 0.22 filter membrane and used for cancer cell treatment. The human cervical cancer cell line HeLa and the human non-small cell lung cancer cell line A549 were cultured in Dulbecco’s modified Eagle’s medium with 10% fetal bovine serum. The ovarian clear cell carcinoma cell line ES-2 was cultured in McCoy’s 5A medium with 10% fetal bovine serum. The colorectal cancer cell line MC38 and the Cellosaurus cell line NCM460 were cultured in RPMI 1640 medium with 10% fetal bovine serum. All the cell lines were cultured at 37°C in a humidified atmosphere with 5% CO_2_. Cell viability assays were performed using the Cell Counting Kit-8 (CCK-8; APExBIO, America, K1018). After centrifugation, the cells were plated in 96-well plates at 10,000 cells per well, cultured overnight, and treated with different concentrations of the filtered bacterial products. After 24 h, the morphological changes in the cells were inspected by phase-contrast inverted microscopy (Primo Star; Carl Zeiss), and 10 µL of CCK-8 solution was added to each well. The plate was incubated for 1 h at 37°C, and the absorbance at 450 nm was detected using a microplate reader. The optical density values were obtained. The inhibition rates on different cells were calculated by GraphPad Prism 8.0.2 software. Cell survival rate (%) = (A [experimental group] − A [blank])/(A [control group] − A [blank]) × 100%.

### Apoptosis assay

To detect apoptosis, cells were incubated with a culture of BI87 and *S. albidoflavus* DSM 40455^T^ for 24 h. The cells were then harvested, washed twice with 1× cold phosphate-buffered saline (PBS), and resuspended in 195 µL of Annexin V-FITC Binding Solution. We then stained the cells with 10 µL of propidium iodide (PI) and 5 µL of Annexin V for 15 min in a dark environment at room temperature and subsequently analyzed the samples by flow cytometry (NovoCyte 451190832384).

### Cell cycle analysis

After treatment with BI87 and *S. albidoflavus* DSM 40455^T^, all cells were harvested and washed twice with PBS, resuspended in 400 µL of PI staining solution, and incubated at 37°C for 30 min. Then, the mixture was incubated at 4°C for 30 min in the dark. Finally, the cells were analyzed by flow cytometry (NovoCyte 451190832384).

### Whole-genome comparison

The preliminary functional annotation was carried out by eggNOG-mapper v2.1.9 (57) and eggNOG database version 5.0 (58). The COGs, KOs, and GOs were extracted from the eggnog mapper result and counted by functional categories. The secondary metabolite BGCs were predicted using the antiSMASH v7.0 (59) beta online program. Antibiotic resistance genes were predicted using CARD (60).

### Core genome-based phylogenetic analysis

We concatenated the core genes common to the bacterial strains analyzed in this study and conducted comparisons using BLAST with the parameters set at >70% DNA identity and >70% gene length to categorize genes into common genes. Individual orthologous sequences were aligned by the MAFFT program (61). The phylogenetic trees of the genomes were structured using the Neighbor-Joining method (62) in MEGA6 (63) by 1,000 bootstrap replicates. The evolutionary distances were computed using the p-distance method (46).

### Growth conditions and phenotypic characterization

The cultural characteristics of strain BI87 and *S. albidoflavus* DSM 40455^T^ were determined after growing for 7 days at 37°C on ISP 1, ISP 2, ISP 3, ISP 4, ISP 5 (64), R_2_A agar, Gause’s agar media, and nutrient agar (65). The coverslip technique (66) was employed to document the hyphae and spore chains. Morphological characteristics were visualized by using light microscopy (Primo Star; Carl Zeiss) and scanning electron microscopy (Quanta 450; FEI) after 7 days of growth on R_2_A.

Growth tests for pH range were carried out by using nutrient broth adjusted to pH 2–12 at intervals of 1 pH unit with 4 mol/L HCl or 5 mol/L KOH after sterilization. NaCl tolerance was examined on R_2_A medium supplemented with concentrations of 0%–10% at intervals of 1% NaCl (wt/vol) after 7 days of incubation at 37°C. The bacteria were incubated at 4°C, 9°C, 19°C, 28°C, 37°C, 40°C, and 50°CC to determine the optimum growth temperature. Other biochemical properties and enzymatic activities were tested using API ZYM kits (Biome’rieux) according to the manufacturer’s instructions at their optimum temperature. The GEN III MicroPlateTM (Biolog) system was used to detect the oxidation of organic substrates according to the manufacturer’s instructions with incubation at 37°CC. Tests for starch hydrolysis, urea hydrolysis, H_2_S production, nitrate reduction, cellulose hydrolysis, and milk coagulation were carried out according to the methods described by Ruan et al. (67).

### Chemotaxonomic characterization

Cell biomass of strain BI87 and *S. albidoflavus* DSM 40455^T^ for chemical studies was obtained from cultures grown on ISP 2 broth or TSB (trypticase soy broth) at 28°C for 14 days, harvested by centrifugation, and washed with distilled water. Diaminopimelic acid was analyzed by TLC (68), and whole-cell sugars were analyzed by TLC (69). The washed cells (100 mg) were saponified, methylated, and extracted. The fatty acid methyl esters were determined using the standard MIDI procedure (Microbial Identification System, Sherlock version 6.1) (70) and gas chromatography (7890A GC system; Agilent). The resulting profiles were identified using the TSBA6 database, version 6.1. The isoprenoid quinones were extracted, purified, and analyzed as previously described (71). The polar lipid contents were extracted from 100 mg freeze-dried cells, examined by two-dimensional TLC (silica gel 60, 10 × 10 cm), and identified as described by Minnikin et al. (72).

## Data Availability

Sequencing reads were deposited in the NCBI Sequence Read Archive under BioProject PRJNA891902 with accession number CP110079. The whole genome sequence data reported in this paper have been deposited in the Genome Warehouse (GWH) under accession number GWHFHGG01000000. All data generated and reagents are available from the corresponding author on reasonable request.

## SUPPLEMENTAL MATERIAL

Table S1 spectrum.00858-25-s0001.xlsx Numbers of genes in common.

Table S2 spectrum.00858-25-s0002.xlsx Secondary metabolite biosynthesis gene clusters.

Table S3 spectrum.00858-25-s0003.docx Phenotypic properties of strains BI87 and the most related type species *Streptomyces albidoflavus* DSM 40455T.

Table S4 spectrum.00858-25-s0004.docx Fatty acid composition.

ASM does not own the copyrights to Supplemental Material that may be linked to, or accessed through, an article. The authors have granted ASM a non-exclusive, world-wide license to publish the Supplemental Material files. Please contact the corresponding author directly for reuse.
