# Supplementary material for: Streptomyces sp. BI87 from human gut: potent anticancer activities and divergence from known Streptomyces lineages
Source: Microbiol Spectr. 2025 Aug 13;13(10):e00858-25. doi: 10.1128/spectrum.00858-25 (PMC12502529; doi:10.1128/spectrum.00858-25)
Supplement: Table S3 — Phenotypic properties of strains BI87 and the most related type species Streptomyces albidoflavus DSM 40455T. [file spectrum.00858-25-s0003.docx]

Supplementary Table 3. Phenotypic properties of strains BI87 and the most related type species *Streptomyces albidoflavus* DSM 40455^T^

Data obtained from this study. +, Positive; -, negative.

| Characteristics | **BI87** | ***S. albidoflavus*** |
| --- | --- | --- |
| Genotypic: |  |  |
| Genome Size (Mb) | 6795 | 6969 |
| GC Content (%) | 73.50 | 74.77 |
| Phenotypic: |  |  |
| Colony Morphology (on Gause’s synthetic No. 1 media) | brown substrate hyphae and abundant white to grey aerial hyphae | white substrate hyphae and abundant white aerial hyphae |
| Temperature (°C) | 25-40 | 25-40 |
| pH | 6-12 | 5-12 |
| NaCl (%, w/v) | 0-4 | 0-8 |
| Available media |  |  |
| NA | + | + |
| R_2_A | + | + |
| Gauze’s agar | + | + |
| ISP1 | + | + |
| ISP2 | + | + |
| ISP3 | + | + |
| ISP4 | + | + |
| ISP5 | + | + |
| Decomposition of Starch | - | + |
| Urea hydrolysis | + | + |
| Produces H_2_S | - | - |
| Nitrate reduction | + | + |
| Hydrolyzes cellulose | - | + |
| Coagulation of milk | - | - |
| Assimilation of (Biolog GEN III): |  |  |
| Dextrin | + | + |
| D-Maltose | + | + |
| D-Trehalose | + | + |
| D-Cellobiose | + | + |
| Gentiobiose | + | + |
| Sucrose | - | - |
| D-Turanose | + | - |
| Stachyose | - | - |
| D-Raffinose | - | - |
| α-D-Lactose | + | - |
| D-Melibiose | - | + |
| β-Methyl-D-Glucoside | - | - |
| D-Salicin | - | - |
| N-Acetyl-D-Glucosamine | - | + |
| N-Acetyl-β-DMannosamine | - | - |
| N-Acetyl-D-Galactosamine | - | + |
| N-AcetylNeuraminic Acid | - | - |
| α-D-Glucose | + | + |
| D-Mannose | - | + |
| D-Fructose | + | + |
| D-Galactose | - | + |
| 3-Methyl Glucose | - | - |
| D-Fucose | - | - |
| L-Fucose | - | + |
| L-Rhamnose | + | - |
| Inosine | - | + |
| 1% Sodium Lactate | + | + |
| Fusidic Acid | - | + |
| D-Serine | - | + |
| D-Sorbitol | - | - |
| D-Mannitol | + | + |
| D-Arabitol | + | + |
| myo-Inositol | - | + |
| Glycerol | - | + |
| D-Glucose-6-PO_4_ | - | + |
| D-Fructose-6-PO_4_ | - | + |
| D-Aspartic Acid | - | - |
| D-Serine | - | + |
| Troleandomycin | - | + |
| Rifamycin SV | + | + |
| Minocycline | - | - |
| Gelatin | - | + |
| Glycyl-L-Proline | - | + |
| L-Alanine | - | + |
| L-Arginine | - | + |
| L-Aspartic Acid | + | + |
| L-Glutamic Acid | + | + |
| L-Histidine | - | + |
| L-Pyroglutamic Acid | - | + |
| L-Serine | - | + |
| Lincomycin | + | + |
| Guanidine HCl | + | + |
| Niaproof 4 | + | - |
| Pectin | - | - |
| D-Galacturonic Acid | - | - |
| L-Galactonic Acid Lactone | - | - |
| D-Gluconic Acid | + | + |
| D-Glucuronic Acid | - | + |
| Glucuronamid e | - | + |
| Mucic Acid | - | - |
| Quinic Acid | - | - |
| D-Saccharic Acid | - | - |
| Vancomycin | - | - |
| Tetrazolium Violet | - | - |
| Tetrazolium Blue | - | + |
| p-Hydroxy-Phenylacetic Acid | - | - |
| Methyl Pyruvate | - | + |
| D-Lactic Acid Methyl Ester | - | - |
| L-Lactic Acid | - | - |
| Citric Acid | + | + |
| α-Keto-Glutaric Acid | - | - |
| D-Malic Acid | - | - |
| L-Malic Acid | + | + |
| Bromo-Succinic Acid | + | + |
| Nalidixic Acid | + | + |
| Lithium Chloride | + | + |
| Potassium Tellurite | + | + |
| Tween 40 | + | + |
| γ-Amino-Butryric Acid | - | + |
| α-Hydroxy-Butyric Acid | - | + |
| β-Hydroxy-D, L Butyric Acid | - | + |
| α-Keto-Butyric Acid | - | + |
| Acetoacetic Acid | + | + |
| Propionic Acid | - | + |
| Acetic Acid | + | + |
| Formic Acid | - | - |
| Aztreonam | + | + |
| Sodium Butyrate | + | + |
| Sodium Bromate | + | + |
| Enzyme activity (API ZYM) |  |  |
| Alkaline phosphatase | + | + |
| Esterase (C4) | + | + |
| Esterase lipase (C8) | + | + |
| Lipase (C14) | + | + |
| Leucine arylamidase | + | + |
| Valine arylamidase | + | + |
| Cystine arylamidase | + | + |
| Trypsin | + | + |
| α-Chymotrypsin | + | + |
| Acid phosphatase | + | + |
| Naphtol-AS-BI-phosphohydrolase | + | + |
| α-Galactosidase | + | + |
| β-Galactosidase | + | + |
| β-Glucuronidase | + | - |
| α-Glucosidase | + | + |
| β-Glucosidase | + | + |
| N-Acetyl-β-glucosaminidase | - | + |
| α-Mannosidase | + | + |
| α-Fucosidase | - | - |
| Fatty Acids (>10%) | anteiso-C15:0, C16:0, iso-C16:0 and anteiso-C17:0 | iso-C_15:0_, anteiso-C_15:0_ and C_16:0_ |
| Polar Lipid Profile | PE and PME | PE |
| Cell Wall Composition | LL-diaminopimelic acid | LL-diaminopimelic acid |
| Whole-cell Hydrolysates | ribose, xylose | ribose |
| Anticancer Activity: |  |  |
| Activity Against Cell Line HeLa (IC50) | 26.27 µL/mL | - |
| Activity Against Cell Line A549 (IC50) | 75.66 µL/mL | - |
| Activity Against Cell Line ES-2 (IC50) | 24.57 µL/mL | - |
| Activity Against Cell Line MC38 (IC50) | 22.37 µL/mL | - |
| Apoptosis Induction Cell Line HeLa | 9.82% | 4.75% |
| Apoptosis Induction Cell Line A549 | 10.82% | 1.82% |
| Apoptosis Induction Cell Line ES-2 | 9.61% | 3.40% |
| Apoptosis Induction Cell Line MC38 | 15.30% | 5.96% |
| Apoptosis Induction Cell Line NCM460 | 2.76% | 2.67% |
| Cell Cycle Arrest (Phase) | Sub-G1, G2 | G2 |
