# Supplementary material for: Streptomyces sp. BI87 from human gut: potent anticancer activities and divergence from known Streptomyces lineages
Source: Microbiol Spectr. 2025 Aug 13;13(10):e00858-25. doi: 10.1128/spectrum.00858-25 (PMC12502529; doi:10.1128/spectrum.00858-25)
Supplement: Table S4 — Fatty acid composition. [file spectrum.00858-25-s0004.docx]

Supplementary Table 4. Fatty acid composition of strain BI87 and *S. albidoflavus.*

Note: –, composition absent.

| Fatty acid | BI87 | *S. albidoflavus* |
| --- | --- | --- |
| C_8:0_ 3OH | - | 0.43 |
| C_9:0_ | - | 0.80 |
| C_10:0_ | - | 0.24 |
| C_10:0_ 2OH | - | 0.57 |
| iso-C_10:0_ | - | 0.88 |
| C_12:0_ | - | 1.01 |
| iso-C_12:0_ | 0.25 | - |
| iso-C_12:0_ 3OH | 0.13 | - |
| C_13:0_ 3OH/iso-C_15:1_ H | 0.24 | 0.44 |
| iso-C_13:0_ | 0.74 | 0.54 |
| anteiso-C_13:0_ | 0.39 | 0.67 |
| iso-C_13:0_ 3OH | 0.38 | 0.91 |
| iso-C_14:0_ | 3.61 | 2.53 |
| iso-C_15:0_ | 7.61 | 13.73 |
| iso-C_15:0_ 3OH | - | 0.16 |
| anteiso-C_15:0_ | 15.23 | 28.77 |
| iso-C_15:1_ F | 0.30 | 0.70 |
| anteiso-C_15:1_ A | 0.08 | 0.18 |
| C_15:1_ ω6c | 0.05 | - |
| C_15:1_ ω5c | 0.09 | - |
| C_16:0_ | 17.97 | 14.25 |
| iso-C_16:0_ | 25.16 | 5.35 |
| iso-C_16:0_ 3OH | 0.09 | - |
| iso-C_16:1_ I/C_14:0_ 3OH | 1.17 | 0.51 |
| C_16:1_ ω7c/C_16:1_ ω6c | 1.60 | 0.33 |
| C_16:1_ω7c alcohol | 0.37 | - |
| C_16:1_ ω11c | 0.26 | - |
| C_17:0_ | 1.63 | 0.53 |
| iso-C_17:0_ | 3.61 | 8.32 |
| anteiso-C_17:0_ | 11.03 | 9.06 |
| C_17:0_ 10-methyl | 0.12 | - |
| C_17:0_ 2OH | - | 0.21 |
| iso-C_17:1_ω9c | 0.21 | - |
| iso-C_17:1_ω10c | 0.33 | - |
| iso-C_17:1_ω5c | 0.57 | 0.25 |
| anteiso-C_17:1_ A | 0.63 | - |
| C_17:1_ω8c | 0.65 | - |
| C_18:0_ | 0.54 | 3.01 |
| iso-C_18:0_ | 0.36 | 0.46 |
| C_18:2_ω6,9c/ante-C_18:0_ | 0.74 | 0.32 |
| C_18:1_ω7c | 0.27 | 0.36 |
| C_18:1_ω9c | 0.70 | 0.65 |
| iso-C_19:0_ | - | 0.43 |
| anteiso-C_19:0_ | - | 0.41 |
| C_20:0_ | - | 0.29 |
